# Using Twitter (X) to Mobilize Knowledge for First Contact Physiotherapists: Qualitative Study

**DOI:** 10.2196/55680

**Published:** 2024-07-08

**Authors:** Laura Campbell, Jonathan Quicke, Kay Stevenson, Zoe Paskins, Krysia Dziedzic, Laura Swaithes

**Affiliations:** 1 Impact Accelerator Unit School of Medicine Keele University Staffordshire United Kingdom; 2 STARS Education and Research Alliance Surgical Treatment and Rehabilitation Services (STARS) The University of Queensland Queensland Australia; 3 School of Medicine Keele University Staffordshire United Kingdom; 4 Midlands Partnership University NHS Foundation Trust Stoke on Trent United Kingdom

**Keywords:** Twitter, X, social media, first contact physiotherapy, musculoskeletal, knowledge mobilisation, primary care, mindlines, qualitative, physiotherapy, implementation

## Abstract

**Background:**

Twitter (now X) is a digital social network commonly used by health care professionals. Little is known about whether it helps health care professionals to share, mobilize, and cocreate knowledge or reduce the time between research knowledge being created and used in clinical practice (the evidence-to-practice gap). Musculoskeletal first contact physiotherapists (FCPs) are primary care specialists who diagnose and treat people with musculoskeletal conditions without needing to see their general practitioner (family physician) first. They often work as a sole FCP in practice; hence, they are an ideal health care professional group with whom to explore knowledge mobilization using Twitter.

**Objective:**

We aimed to explore how Twitter is and can be used to mobilize knowledge, including research findings, to inform FCPs’ clinical practice.

**Methods:**

Semistructured interviews of FCPs with experience of working in English primary care were conducted. FCPs were purposively sampled based on employment arrangements and Twitter use. Recruitment was accomplished via known FCP networks and Twitter, supplemented by snowball sampling. Interviews were conducted digitally and used a topic guide exploring FCP's perceptions and experiences of accessing knowledge, via Twitter, for clinical practice. Data were analyzed thematically and informed by the knowledge mobilization mindlines model. Public contributors were involved throughout.

**Results:**

In total, 19 FCPs consented to the interview (Twitter users, n=14 and female, n=9). Three themes were identified: (1) How Twitter meets the needs of FCPs, (2) Twitter and a journey of knowledge to support clinical practice, and (3) factors impeding knowledge sharing on Twitter. FCPs described needs relating to isolated working practices, time demands, and role uncertainty. Twitter provided rapid access to succinct knowledge, the opportunity to network, and peer reassurance regarding clinical cases, evidence, and policy. FCPs took a journey of knowledge exchange on Twitter, including scrolling for knowledge, filtering for credibility and adapting knowledge for in-service training and clinical practice. Participants engaged best with images and infographics. FCPs described misinformation, bias, echo chambers, unprofessionalism, hostility, privacy concerns and blurred personal boundaries as factors impeding knowledge sharing on Twitter. Consequently, many did not feel confident enough to actively participate on Twitter.

**Conclusions:**

This study explores how Twitter is and can be used to mobilize knowledge to inform FCP clinical practice. Twitter can meet the knowledge needs of FCPs through rapid access to succinct knowledge, networking opportunities, and professional reassurance. The journey of knowledge exchange from Twitter to clinical practice can be explained by considering the mindlines model, which describes how FCPs exchange knowledge in digital and offline contexts. Findings demonstrate that Twitter can be a useful adjunct to FCP practice, although several factors impede knowledge sharing on the platform. We recommend social media training and enhanced governance guidance from professional bodies to support the use of Twitter for knowledge mobilization.

## Introduction

Digital social networks are an evolving way for health care professionals and researchers to quickly find, share, and use knowledge [1-3]. Although Facebook, YouTube, WhatsApp, Instagram, and TikTok are the world’s most used social media platforms [4], the open, public arena of Twitter (now known as X) offers health care professionals access to many diverse sources of knowledge. Twitter is a popular, free-to-use forum for communication amongst the public and between health care professionals and patients [5] and can offer health care professionals additional insight and understanding into, for example, patient narratives, which are often hidden behind private support groups on platforms such as Facebook, and rarely mentioned on professional platforms such as LinkedIn. The use of Twitter involves users posting short messages that can rapidly be commented on, liked, or reposted by other users worldwide, providing health care professionals with access to a high volume of succinct knowledge in various formats, for example, images, text, videos, and links, unlike other platforms that may focus solely on images or videos. Yet, little is known about how health care professionals find, adapt, use, and share knowledge from Twitter to inform evidence-based clinical practice.

Twitter has been postulated as a solution to reduce the evidence-to-practice gap—the delay between the production of health care research knowledge and its uptake in clinical practice [6]. Knowledge mobilization (KM) seeks to accelerate and facilitate the dynamic nature of creating, sharing, and adapting research and other forms of knowledge across professional, public, and organizational domains, to the point where it can be most useful for stakeholders [7,8]. KM is a social process that acknowledges real world contextual complexities.

One approach to mobilizing knowledge among health care professionals is through enhancing mindlines [8]. Mindlines are “internalised, collectively reinforced and often tacit guidelines in the head” that underpin rapid clinical decision-making in highly pressurized, complex environments [9]. They are informed by a diverse combination of explicit and tacit individual and collective knowledge, experience and storytelling, clinical training, reading, and understanding of local contexts, among other sources, such as social media. Individual and collective mindlines are continuously constructed, challenged, and reinforced through informal conversation with peers [10] and are actively tried, tested, and contextualized in the real world [9]. The mindlines model can be used to study how people use social processes to interact with each other to find, adapt, use, and share knowledge on both an individual level and collectively, and it can be applied to the world of social media.

A recent UK policy push to increase the provision and remit of multispecialty teams and reduce pressures in primary care has seen the introduction of specialist musculoskeletal (MSK) first contact physiotherapists (FCPs) [11]. FCPs work in general practice to diagnose and treat MSK symptoms without patients needing to see their general practitioner (or family physician) [12]. In contrast to traditional physiotherapy departments, most FCPs typically work as the sole FCP in a general practice away from peers [13,14], and many split their time working across multiple practices, services, or additional roles. Within the primary care context of the rapid evolution and implementation of FCP, variation in practice and training [15], and feelings of uncertainty about the FCP role [14], little is known about where FCPs obtain knowledge for clinical practice outside of their teams.

The aim of this study was therefore to explore how Twitter is, and can be, used to mobilize knowledge to inform FCP clinical practice. The mindlines model is used as a lens through which to explore and understand the social processes associated with how FCPs use different types of knowledge from Twitter to inform their mindlines. Findings from this study could support FCPs in using Twitter to find, adapt, and share knowledge for clinical practice and have implications for the way that social media use is governed by professional bodies, such as the National Health Service (NHS) Integrated Care Systems (ICSs) and the Care Quality Commission.

## Methods

### Overview

A qualitative interview study was conducted. Purposive sampling was used to recruit English NHS MSK FCPs based in general practice with a range of employers (NHS hospital and community trusts, NHS service providers, Primary Care Networks) who used Twitter for professional purposes. To gain a breadth of perspectives, non–Twitter users (NTs) were also recruited. Participants were recruited via professional networks (including Versus Arthritis charity clinical networks, Keele University Allied Health Professionals Critically Appraised Topic Group [16], the Chartered Society of Physiotherapy FCP mailing list, Keele University Impact Accelerator Unit [17], health care professionals training networks, national and local FCP networks, and via Twitter itself). This was supplemented with snowball sampling to identify potential FCP participants via extended professional networks unknown to the research team. Participants were included in the study if they were currently employed as an MSK FCP in NHS primary care in England. Recruitment continued until inductive thematic saturation was achieved [18].

The COVID-19 pandemic necessitated the use of Teams (Microsoft Corp) to conduct semistructured interviews virtually. Two pilot interviews were carried out with FCPs in the study Stakeholder Advisory Group (SAG). With informed consent, interviews were conducted by LC (Knowledge Broker, female, with training in KM research and practice and qualitative research). Topic guides were iteratively modified as interviews took place and new findings emerged. Final topic guides are included in Multimedia Appendix 1. A digital voice recorder was used to capture the interviews, which were transcribed verbatim and pseudonymized by LC. Participants were assigned codes according to the order in which they were interviewed (P01, P02, etc.) and whether they were a Twitter user (T) or NT. Video content was not recorded. The reporting of this study is in line with the COREQ (consolidated criteria for reporting qualitative research) checklist [19] (Multimedia Appendix 2).

### Data Analysis

Data analysis was iterative and largely inductive, informed by the principles of reflexive thematic analysis [20,21]. Memos, decision logs, debriefing, regular meetings with the research team, a reflexive diary, and a researcher positionality statement maintained critical reflexivity throughout the study, allowing the findings to reflect the research question, aims, and objectives and not the bias of the researcher (Multimedia Appendix 3). Furthermore, analysis decisions were regularly discussed with the multidisciplinary SAG and KM experts.

Coding was conducted by LC, with double coding of a subset of transcripts by LS and JQ. Full details of the analysis steps taken are described in Multimedia Appendix 4.

### Stakeholder Involvement

A multidisciplinary SAG consisting of patients and the public, academics, FCPs, physiotherapists, a marketing professional, and KM practitioners was convened at the start of the study to ensure relevance to the research topic, to develop, test and refine the interview topic guides and to inform interpretation of the data. SAG meetings were held via a videoconferencing platform (Teams).

Patient and public involvement and engagement was embedded throughout the study, and reporting is aligned to GRIPP (Guidance for Reporting Involvement of Patients and the Public) 2–Short Form [22]. Three public contributors with varying experience of MSK conditions, Twitter, and KM [23,24] were members of the SAG. They coproduced the topic guides, and informed interpretation of data, and codeveloped the plain language summary. Public contributors were reimbursed for their time in line with the National Institute for Health and Care Research’s (NIHR’s) Public Involvement Standards [25]. Further details are included in Multimedia Appendix 5.

### Theoretical Underpinning

The mindlines model [9,10,26] was chosen as an additional lens through which to interpret the data from a KM perspective. This provided rich, contextualized insights into the social processes behind FCPs’ use of knowledge from Twitter in clinical practice.

### Ethical Considerations

Ethical approval was obtained from Keele University’s Faculty of Medicine and Health Sciences Research Ethics Committee 28.10.21 (REC reference MH-210199) with no conditions. See Multimedia Appendix 6. Participants were fully informed through the provision of a participant information sheet before consent was taken and given the option to opt out at any time up to 2 weeks following the interview date. Interview transcripts were pseudonymized so that participants could not be identified. Participants took part on a voluntary basis.

## Results

A total of 25 FCPs expressed an interest in the study. One potential participant did not meet the eligibility criteria (not based in England) and 5 did not respond after receiving the study information. In total, 19 MSK FCPs (Ts, n=14; NTs, n=5), from 6 different geographical regions of England consented to be interviewed via a videoconferencing platform (Teams) between November 2021 and February 2022. Interviews lasted between 22 and 92 minutes. Brief participant characteristics are included in Table 1.

**Table 1:** Participant characteristics.

Characteristic		Participants, n
**Recruitment**		
	Keele University networks	3
	Twitter	5
	Snowball sampling—extended FCP^a^ networks	10
	Tagged^b^ by colleague on Twitter	1
**Gender**		
	Male	10
	Female	9
**Employment**		
	NHS^c^—Foundation Trust	11
	NHS—Integrated Care Trust	2
	NHS—Community musculoskeletal service	2
	NHS—Clinical Commissioning Group	1
	NHS and private practice	1
	Social enterprise organization providing NHS community services	2
**Additional roles**		
	NHS leadership	4
	Split posts	15
	Full-time FCP	4 (2 split across practices)
**Experience**		
	Qualified—physiotherapy	Between 5 and 24 years
	FCP role	Between 3 months and 3 years
**Twitter user**		
	Yes	14
	No	5

^a^FCP: first contact physiotherapist.

^b^Tagged on Twitter—when a Twitter user identifies someone else to bring information in a post to their attention.

^c^NHS: National Health Service.

**Figure 1:**
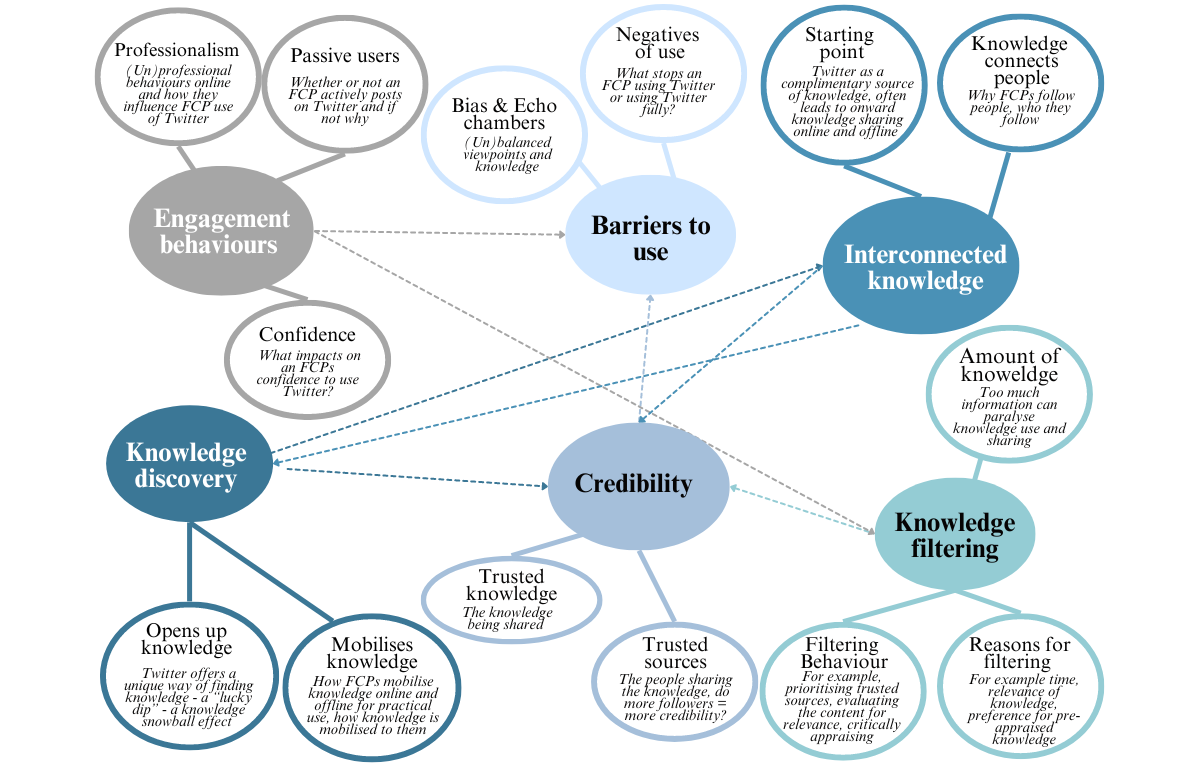
Initial thematic map with themes, subthemes, and relationships. FCP: first contact physiotherapist.

**Figure 2:**
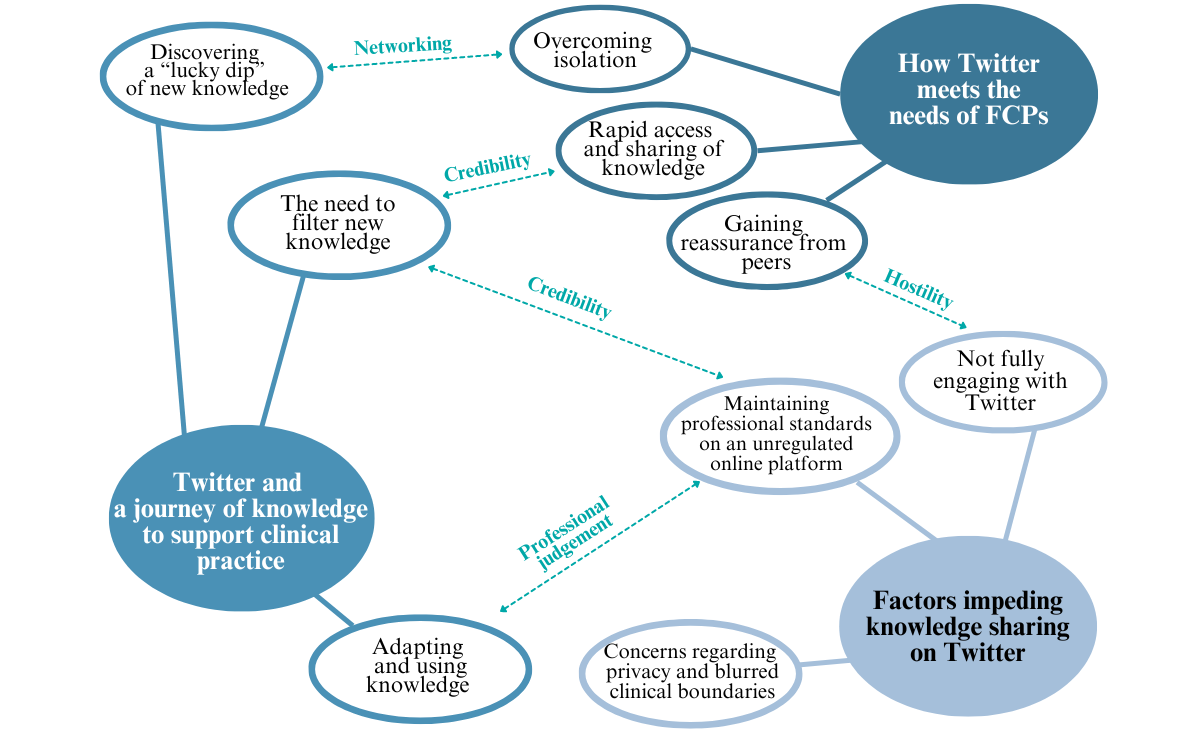
Final thematic map with themes, subthemes, and relationships. FCP: first contact physiotherapist.

**Figure 3:**
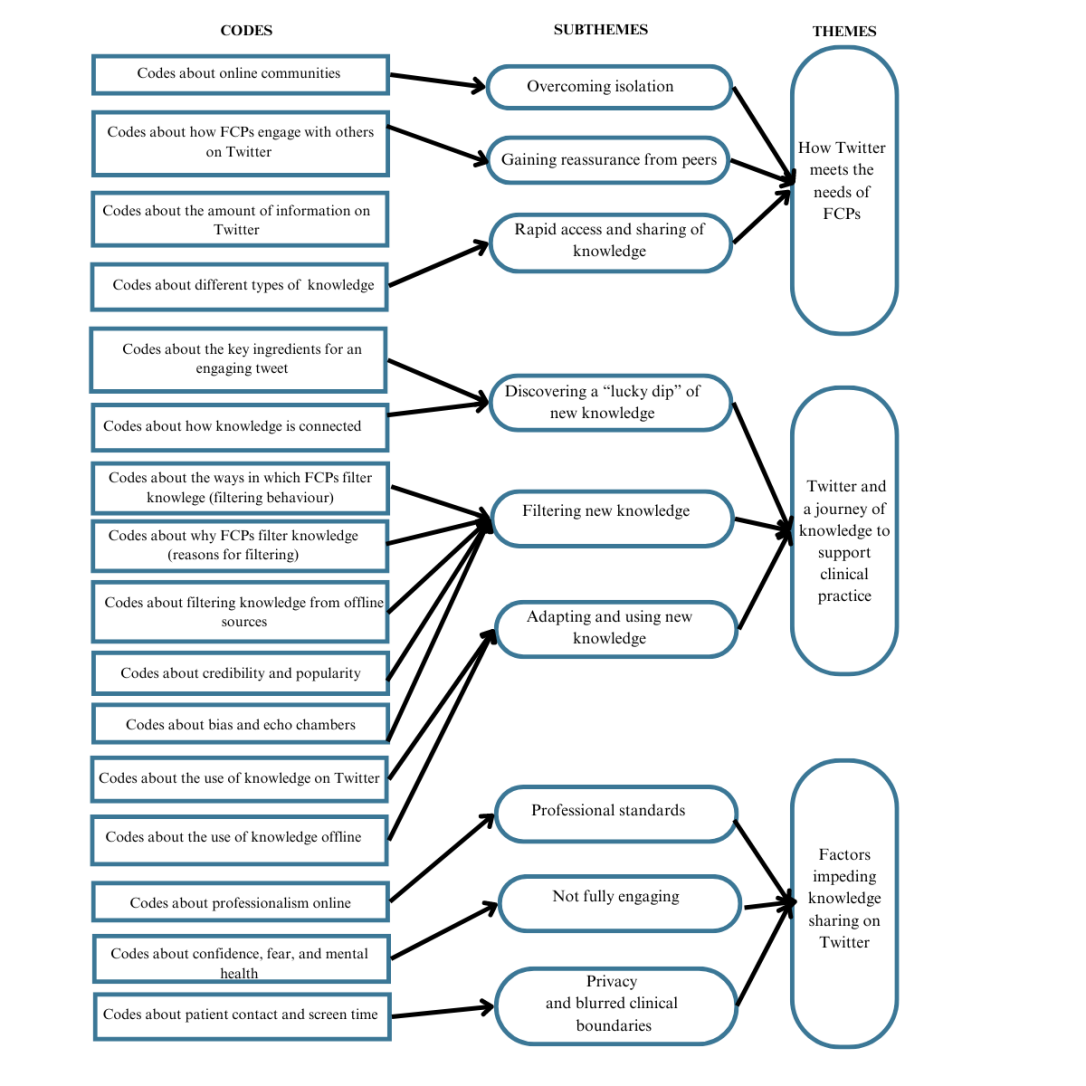
Overview of data structure. FCP: first contact physiotherapist.

Thematic maps were used to support data analysis by visualizing the relationships between codes, themes, and different levels of themes (Figures 1 and 2). Figure 1 outlines an initial set of themes and subthemes and the relationships between them. These themes and subthemes were then further refined and interpreted through iterative discussion between the study team and the SAG, as initial themes overlapped and were not clear. Figure 2 shows the final themes and subthemes. The boundaries between these were clarified, and the core concepts for each theme and subtheme were defined by the study team in the context of the overall narrative of the data. Subthemes were used to provide more interpretive depth. Figure 3 provides an overview of the coding structure.

Three key themes were identified: (1) How Twitter meets the needs of FCPs; (2) Twitter and a journey of knowledge to support clinical practice; and (3) factors impeding knowledge sharing on Twitter.

A description of each theme and subtheme and supporting quotes are presented below.

### Theme 1: How Twitter Meets the Needs of FCPs

#### Overcoming Isolation

Several participants described feeling isolated from other FCPs working as the sole therapists in primary care practice. Additional to this, fragmented working patterns between roles and sites, a lack of supervision and mentorship, remote working, and constantly changing policies and guidance contributed to feelings of professional and personal loneliness:

It’s hard, you don’t get the same interaction [...] You can feel a bit isolated.

P07 T

Some participants used Twitter more often after moving from being part of a unified secondary care team to an isolated primary care practice:

I didn’t use Twitter as much in rheumatology as I do now and I think it’s because the more remote you are, I mean, I work on my own in a room.

P06 T

Twitter was indicated as a potential solution to overcome professional isolation, offering social networking with others to “promote conversation” (P09 T). Additionally, it was thought to provide an opportunity to learn, share best practices and access this study for evidence-based care often hidden behind journal paywalls:

‘What’s the latest evidence on this’ or ‘What’s been released recently?’ or ‘What discussions have been going on about this?’ I find it [Twitter] really useful, really helpful for that.

P05 T

#### Rapid Access and Sharing of Knowledge

Rapidly changing knowledge could be accessed quickly and easily via Twitter within the time-pressured context of FCP roles to “keep up to date with current thinking” (P08 T) around research and clinical guidelines for best patient care, with short character limits of tweets conveying key, succinct knowledge:

I think with Twitter […] they give the kind of pertinent points of a research study or something that’s easier to remember, more easily digestible and the kind of key take home messages.

P13 T

In contrast, research databases were described as “old fashioned” (P01 T).

Twitter offers many different types of knowledge and sources, allowing participants to be able to “keep on board with lots of things all in one place” (P11 T) and to share knowledge with patients and peers. Yet, this was also described as overwhelming and time-consuming to find, engage with, and share relevant knowledge.

#### Gaining Reassurance From Peers

Participants used Twitter to get “the feel of what other people are thinking and saying about different things” (P02 T), which gave them reassurance in their professional practice:

So there can be that reassurance that you look at a case study [on Twitter] and based on what everyone else is saying you think, oh yeah, well I'd have done that.

P11 T

Conversely, one participant highlighted that Twitter promoted feelings of professional insecurity when faced with not keeping up with all new knowledge shared within digital FCP social networks; however, the majority felt it was valuable for reassurance around wider contextual policy concerns, continuing professional development (CPD), and the development of the novel FCP role:

Is it only me that’s stressed about it or is everybody thinking the same thing? […] it’s that kind of support, even though you don’t get factual information, it’s people talking about things and they’re going through the same things like we’re going through.

P14 T

Seeing tweets from opinion leaders who were “on the same wavelength” (P06 T) and the visibility of senior staff in leadership roles using Twitter to offer guidance and inspiration was thought to be important, yet participants did not see themselves as having sufficient “status” to offer reassurance themselves or to share their own knowledge and experiences:

I think I would probably need to tag some big names so that more people saw it and get other people to retweet it and things like that, because I think me on my own probably wouldn’t reach very far.

P02 T

Twitter’s “always on” culture accentuated a perceived expectation that participants must use the social network “to be seen to be in the loop” (P08 T). Guilt around not having time to promote knowledge themselves and anxiety regarding missing out on knowledge were also acknowledged by NTs:

They may be getting loads of information and knowledge through Twitter that actually I could be getting and missing out on, and if that were the case, then I would want to know.

P15 NT

### Twitter and a Journey of Knowledge to Support Clinical Practice

#### Discovering a “Lucky Dip” of New Knowledge

Participants did not purposefully look for specific knowledge on Twitter; instead, they browsed through their feeds to discover new knowledge from a range of multidisciplinary sources. Knowledge of value included new digital social networks, research evidence, clinical scenarios, imaging, guidelines, training opportunities, expert opinions, and service developments:

An example would’ve been that I don’t think I’d have known about the chronic fatigue NICE guidelines coming out if I hadn’t have seen somebody share that on Twitter.

P16 T

Conversely, participants also described anxiety around a lack of control over what knowledge they would see and expressed concerns about losing time when scrolling through Twitter:

I think you could get sucked down the rabbit hole of just endlessly scrolling and how much use would that actually be?

P02 T

Fellow FCPs, physiotherapists, governing bodies, opinion leaders, and researchers were followed because they were also followed by people in their digital social networks, conceptualized by one participant as “a bit of a chain really” (P09 T). Tweets containing pre-packaged, trustworthy content, graphics, videos, and simple concise messages that were of use for clinical practice were agreed to be most engaging for participants:

The ones that I really like are ones that have a little picture or graphic attached to it […] things that you can pass onto patients as well so, patient friendly information, there’s a few things that you can just print off and put in the clinic room and bits like that and that’s really helpful.

P13 T

#### The Need to Filter New Knowledge

There was an awareness of potential clinical misinformation and a subsequent need to filter both content (tweets) and sources (people posting) for credibility; yet, this was not done routinely or systematically. Although web-based debate was seen as informative, comments on tweets were mostly ignored unless backed up by research evidence; however, one participant talked about these acting as a mini “critical appraisal (P01 T)” system. There was concern that physiotherapists with more followers would be automatically seen as more credible, with the heightened power of physiotherapy influencers seen as potentially unethical.

For some, echo chambers (when the same ideas and opinions are repeated, reinforcing beliefs and encouraging bias) were believed to be a risk due to potentially biased knowledge being shared within the small digital physiotherapy community, Twitter’s algorithms, and FCPs working in isolation:

There is a big risk as clinicians as we develop to one school of thought because we follow the people we agree with only. And then we end up causing, not harm but possibly missing out on a lot of good information.

P12 T

In contrast, others felt the amount of knowledge available on Twitter plus debate and discussion in this “open forum” (P07 T) for digital communication reduced the risk of echo chambers and bias.

Sources that were considered credible were professional, respected national bodies such as the Chartered Society of Physiotherapy, academia, or the NHS:

The national bodies are pretty robust because a lot of that has already been filtered, so I’m aware that’s already been reviewed before it’s been put out so that’s pretty trustworthy.

P01 T

#### Adapting and Using Knowledge

Participants did not use explicit knowledge (such as clinical guidelines or research findings) found on Twitter directly; instead, they combined this with their own experience and more tacit knowledge, discussing the importance of “the other bits beyond MSK that you need in this role” (P09 T), namely the tacit understandings of local processes and professional culture*.* Explicit knowledge from Twitter was included in CPD, training sessions, clinical case discussions, and shared through emails and WhatsApp groups. However, all participants who used Twitter adapted explicit knowledge to local contexts or summarized it in other formats:

When the MSK standards came out the other week, the first place I saw them was on Twitter. So I read them, summarised them, put a little PowerPoint presentation together for the whole service and said, look, I don’t anticipate everyone’s going to spend time reading 72 pages of this document but these are the key points.

P18 T

NTs interacted with knowledge on other social media platforms, such as LinkedIn, in similar ways:

Someone shared something on LinkedIn […] that summarised everything we talked about for three hours. So I immediately saved that, printed it off and I use it on a daily basis.

P03 NT

Many participants discussed seeing clinical case studies posted and discussed on Twitter, describing that these widened “clinical reasoning in terms of differential diagnosis” (P16 T) and enhanced their conceptual understanding of multiple clinical conditions.

### Factors Impeding Knowledge Sharing on Twitter

#### Maintaining Professional Standards on an Unregulated Web-Based Platform

All participants who used Twitter described witnessing what they perceived to be unprofessional behavior within the digital physiotherapy community. They spoke about observing “heated arguments and swearing” (P02 T), a “toxic environment” (P08 T, P02 T, and P18 T), and “inflammatory comments” (P16 T), which made them feel sad, embarrassed, and concerned about the detrimental “issue with professionalism on Twitter” (P12 T). Even NTs were familiar with aggression on the platform:

((Names physiotherapist)) is bashing them saying, research doesn’t suggest this works or that. But he doesn’t really share any of his case studies, he’s just bashing this guy because he does something which is a bit different.

P17 NT

Participants described having a professional responsibility to maintain standards on Twitter, particularly when posting clinical content to a public audience, and to not give clinical advice to members of the public. Although acknowledging that “free speech is important” (P02 T), participants suggested ways in which professional behavior needed to be “regulated” and “policed” (P01 T), and several suggested that the profession would benefit from social media training at the undergraduate, postgraduate, and CPD levels, with particular support for students and newly qualified practitioners.

#### Not Fully Engaging With Twitter

Despite being experienced and knowledgeable health care professionals, witnessing hostilities within the web-based physiotherapy community resulted in participants who used Twitter feeling anxious to share knowledge on the social network themselves:

You’re doing something, or managing a service a certain way, or behaving in a certain way with your patients, and somebody disagrees, it can be quite a volatile place.

P01 T

This led to some participants preferring to use the more private direct message function over public tweeting, and the majority defining themselves as lurkers:

I don’t post anything mainly because I’m not going to say the wrong thing and get loads of abuse, I’ll just quietly lurk and look at what other people say.

P02 T

Reasons for lurking were multifaceted and included fear of making mistakes, a lack of time to accurately and actively share knowledge and the use of personal rather than organizational accounts. Several participants had positive attitudes toward the benefits of active engagement on Twitter; yet, they avoided giving knowledge in favor of taking it instead. Despite these passive, lurking behaviors, participants actively used knowledge from Twitter offline to inform their clinical practice, contributing to contextual, tacit understanding and “facilitating good conversations with teams” (P13 T):

I think it’s good to know what the conversations being had are and perhaps what the kind of arguments both sides are [on Twitter] but I think ultimately it’s more helpful to have the discussion with the colleagues and people that you’re working with and also, the population that you’re working with to see what’s going to work best for your team, what’s going to work best for the population that you serve.

P13 T

#### Concerns Regarding Privacy and Blurred Clinical Boundaries

Participants described how Twitter blurs the boundaries between professional and personal lives and expressed concern about 24/7 patient contact on digital platforms potentially breaching these boundaries:

That’s my time. I don’t want you impinging, I will see you in my clinic when it’s your appointment, but I don’t want you having access, to be in my thoughts and what I’m doing when I’m not at work. Because that’s not the deal.

P02 T

Further concerns around negative comments and complaints from patients on Twitter were discussed, as well as a fear for privacy and personal security. One participant discussed how a patient had found and used a family image from her social media for his screensaver and how this had directly affected the way she uses Twitter:

I’m not really active at putting stuff on [Twitter], from a security point of view. But I do say this to staff, cos they put all their family information, their kids, their full name, you can see their house. It is really easy for people to be found then.

P07 T

Only one participant described a positive experience of engaging with patients on Twitter, although maintaining professional boundaries in this situation was also acknowledged:

I had a lady who wanted to run a half marathon and I discharged her six months, she’d got a training programme, she just contacted me on Twitter to say she’d done it and that’s great, [...] equally we just have to be aware of those boundaries a little bit as well.

P18 T

## Discussion

### Principal Results and Comparison With Prior Work

To our knowledge, this semistructured interview study is the first to explore how FCPs use Twitter as a source of knowledge to inform their clinical practice and contributes to the growing literature on the use of social media for digital KM. This study shows that Twitter provides FCPs with rapid access to succinct knowledge, networking opportunities, and peer reassurance regarding clinical cases, evidence, and policy. It demonstrates the need for FCPs to understand how to find appropriate knowledge on Twitter, filter it for credibility, and adapt it for in-service training and clinical practice. This study highlights many factors impeding FCPs from sharing knowledge on Twitter and their consequent lack of confidence to actively participate on Twitter.

Participants reported that the functionality of Twitter supports their time pressured and fast paced roles by offering rapid access to brief summaries of diverse knowledge from diverse sources. This is particularly useful since FCPs work in a context where their isolated role is weighted toward rapid clinical assessments and access to current knowledge, training, and CPD can be difficult [15]. This reflects commentary noting Twitter as a valid way of keeping up to date with research that is inaccessible behind paywalls and without formally searching research databases [2,27]. FCPs reported being most likely to engage with tweets containing prepackaged knowledge and salient points of research evidence. This aligns with the emergence of knowledge translation tools such as actionable nuggets [28] and clinical knowledge summaries [29] and recent commentary discussing how Tweetorials (a collection of tweets that aim at educating users who engage with them) and tweet threads (a series of connected posts from one person) are useful to keep up to date with research findings [30]. Visual posts were reported by participants as being most engaging, aligning with a 2019 systematic review that found that health care professionals believe infographics reduce the time burden of reading full texts [31].

Social connection with peers, researchers, and opinion leaders through following, retweeting, and liking posts was seen as important in the FCP context, as most work in isolation away from FCP peers and many split their time working across multiple practices [13-15]. Participants described feeling reassured when reading tweets relating to clinical questions, evidence, and constantly changing policy and guidance for the FCP role; findings are useful in light of recent interviews with FCPs exploring common feelings of uncertainty regarding the FCP context and role [13,14]. Many expressed a fear of missing out on something on Twitter, perhaps echoing this uncertainty. Accessing wider perspectives and opinions helped to inform their own clinical decisions, highlighting the role that Twitter plays in cross-disciplinary knowledge sharing [32]. Furthermore, these social connections facilitated CPD and learning for FCPs, findings consistent with a systematic review highlighting Twitter as a vehicle for education and training amongst frontline clinical peers for professional development [1].

A key barrier to FCPs sharing knowledge on Twitter was the perceived hostile environment, which impacted their confidence to exchange knowledge and opinions on the platform (irrespective of their clinical experience). FCPs did not feel safe in this open public forum, believing they would encounter intimidation and toxicity, consistent with literature highlighting unprofessional behavior on Twitter as a concern for health care professionals using it [1,2,33,34]. This barrier to use was consistent with why some participants did not use Twitter. Instead, FCPs were happy to take knowledge from Twitter and adapt and use it in different contexts and communities offline, feeling more comfortable sharing knowledge in more familiar team meetings or with colleagues in person. These findings complement the analysis of 8711 web-based communities, which found that about 90% of digital community members are “lurkers” [35], and case studies, which found lurkers to be more active in sharing information offline [36]. FCP NTs welcomed the knowledge brought to team discussions by FCP Ts. FCP roles often span role and organizational boundaries, offering a unique opportunity to share knowledge, skills, and ideas from Twitter across other networks [37], such as members of the wider primary care practice, primary care networks, ICSs, and FCP training networks. Although there is guidance for the broader physiotherapy profession to support the use of social media [38], there is no known existing guidance that is specific to FCPs, whose contexts, demands, and working environments differ from those of other physiotherapists.

Although participants reported the rapid access to diverse knowledge on Twitter as useful for clinical practice, at the same time they described the volume of information as occasionally overwhelming and requiring filtering for relevance. This echoes commentary in the field of medical education that accessing information on Twitter can resemble “drinking from the firehose” [2], which can conversely take up more valuable time. Additionally, participants were concerned about the risk of bias, echo chambers, privacy, blurred personal boundaries, and misinformation, often cited as pitfalls of social media for health care professionals [2,33,34,39,40]. FCPs in this study did not routinely or systematically appraise knowledge on Twitter for credibility and automatically trusted knowledge posted by authoritative national bodies and academia, findings that were similar to a cross-sectional survey of 203 physiotherapists and students in New Zealand that explored their use of electronic information for CPD [40].

### How Key Findings Relate to the Mindlines Model

Applying the mindlines model to our analysis provided deeper insight into how knowledge from Twitter was shared, discussed, adapted, and used on both individual and collective levels. Knowledge from Twitter was frequently taken into offline discussions and training, allowing FCPs the opportunity to collectively combine, discuss, challenge, and make sense of knowledge to adapt it to fit local contexts, becoming what has been described in the literature as “Knowledge-in-Practice-in-Context” [9,41]. The reported hostile environment on Twitter reinforces why FCPs preferred not to contribute to web-based discussions and debate but to take knowledge offline to inform CPD and clinical practice. The knowledge gleaned from Twitter therefore did enhance FCP mindlines, aligning with KM literature, which outlines trusted “safe spaces” as a necessary prerequisite for mindlines to develop [8]. Conversely, misinformation on Twitter could pose a risk of inaccurate information becoming internalized into individual FCP mindlines. Subsequently, through ongoing knowledge sharing, “mindlines can spread collective folly” [9] and move into the collective FCP thought. This risk further explains why FCPs instinctively prefer to sense-check knowledge found on Twitter with trusted colleagues in face-to-face, offline contexts.

FCPs described taking multiple forms of knowledge they found on the platform and combining them with existing knowledge to make decisions. In this respect, Twitter resembles the insertion of knowledge into mindlines observed by Gabbay and le May in face-to-face contexts [25]. This study has shown how FCP informal debate and experience sharing on Twitter supported both FCPs explicit clinical knowledge and a more nuanced tacit contextual understanding of FCP policy, norms, and role expectations. This echoes research that determined that a blend of explicit and tacit knowledge is important for mindline development, both on digital social networks and offline [26,42]. This contemporary study has shown how knowledge sources informing FCP mindlines are moving beyond in person conversations and expanding to include digital social networks.

### Implications for Research and Practice

As multispecialty teams in primary care continue to evolve [11], so too do the ways in which health care professionals such as FCPs access knowledge for clinical practice [1,34]. This study demonstrated how the use of Twitter in health care can support FCPs to be more informed by offering access to many different types of new knowledge and connections to peers. However, specific guidance for its use should be considered for implementation by local NHS MSK ICSs, professional bodies, and the Care Quality Commission. Further work is needed to cocreate this guidance with stakeholders based on the findings from this study. This should include the development and implementation of further social media training for FCPs to avoid inappropriate professional conduct and empower FCPs to use social media in a responsible and effective way for learning. Training could offer ways to overcome the potential barriers to the use of Twitter for KM highlighted by this study. Specifically, it should include how to access relevant information, how to identify misinformation and evaluate tweets, the importance of maintaining professional standards and behaviors, and ways of maintaining privacy if desired. In addition, guidance should also include increased visibility of senior leadership and governance for physiotherapists regarding digital professionalism, which would also contribute to psychological safety on digital social networks.

For people seeking to communicate and mobilize health knowledge, Twitter could play a role in developing 2-way relationships to share knowledge across professional, public, and organizational boundaries and between research and practice. Researchers and knowledge mobilisers must consider the drivers and challenges of the FCP community when mobilizing knowledge using Twitter and take into account the preferred ways in which knowledge is exchanged. Given that increased numbers of health care professionals, such as FCPs, are using social media to access knowledge, implementation, and KM strategies should include the use of social media. Based on findings from this study, Tweet-ups (when users can join a chat around a specific topic using a hashtag at a certain time) or Tweetorials are potential practical examples of how this could be done within the FCP community. Using visual tweets and infographics on Twitter is recommended to effectively engage FCPs and communicate knowledge. This study has shown how KM is complex and messy [43] and spans both digital and offline spaces. Knowledge mobilisers should therefore supplement the use of Twitter as a knowledge source with face-to-face means such as discussions or communities of practice—an established strategy for mobilizing knowledge [44].

Further research exploring the use of social media amongst other professional groups would develop a fuller picture of the role that Twitter plays in health care practice, and questions still remain about what makes health care professionals frequent tweeters and how those who are less confident can be supported to use this digital social network effectively. Future work to address the ways in which social media may be used to mobilize knowledge between health care professionals, patients, and the public is also needed.

### Limitations

Strengths of this work include the robust, theory-informed approach to reflexive thematic analysis to develop final themes and actionable outcomes [45]. The use of purposive and snowball sampling enabled the recruitment of a broad range of FCPs from varying employment and geographical backgrounds. Partnership working and coproduction in the SAG was a particular strength of the study, enabling the identification of areas for discussion in the topic guides not previously considered, for example, the ways in which patients may engage with FCPs on Twitter.

A potential limitation is that data collection took place before Twitter changed ownership and rebranded as X, changing the context in which this study was based. Recruitment via physiotherapy networks and Twitter risked potential response bias, however, this was mitigated by both purposive and snowball sampling methods to recruit a broad range of perspectives (including NTs). Although this study focused on one health care professional group, potentially impeding the transferability of results to other groups, the findings illustrate key issues likely to be comparable to those of others working in isolation, such as general practice nurses [46], general practitioners, community pharmacists, or practice managers. Furthermore, there is a limit to how well social media posts can comprehensively represent all knowledge and information. A number of authors researching in this area have identified temporal and geographic biases in tweets [47,48], which may influence the knowledge accessed by this study’s participants through Twitter. While some findings may be applicable to other countries, this study was limited to the context of English NHS primary care. The study purposefully interviewed 5 NTs, which was enough to achieve theoretical saturation within the aims of this study. Semistructured interviews risk response bias and interviewer bias [49]; however, participants spoke openly about both positive and negative accounts of using Twitter, and researcher reflexivity was used throughout the study. Data regarding self-reported ethnicity was not collected—it is recognized that this would have been useful to describe the diversity of the sample. Finally, the study was conducted during the COVID-19 pandemic, which necessitated the use of Teams rather than face-to-face interviews. This has been suggested to stifle rapport building [50], but was convenient for busy clinicians and has been shown to produce similar richness and quality of data as traditional face-to-face methods [51,52].

### Conclusions

To the best of our knowledge, this is the first study to explore how Twitter is and can be used to mobilize knowledge to inform FCP clinical practice through the exploration of FCP's perceptions and experiences of using the platform. It illustrates how Twitter can meet the knowledge needs of FCPs by providing rapid access to succinct knowledge, networking opportunities, and professional reassurance. The novel use of the KM model mindlines as a theoretical lens provided a deeper understanding of the journey of knowledge exchange from Twitter to clinical practice by describing how FCPs access, adapt, and share diverse knowledge with peers in digital and offline contexts. Although several factors impede knowledge sharing, we recommend social media training and enhanced governance guidance from professional bodies to support its potential to have a pivotal role in KM.

## Appendix

Multimedia Appendix 1 Interview topic guides.

Multimedia Appendix 2 COREQ (consolidated criteria for reporting qualitative research) checklist.

Multimedia Appendix 3 Positionality statement.

Multimedia Appendix 4 Analysis steps.

Multimedia Appendix 5 Patient and public involvement.

Multimedia Appendix 6 Ethics decision.

## Abbreviations

COREQ: consolidated criteria for reporting qualitative research
CPD: continuing professional development
FCP: first contact physiotherapist
GRIPP: Guidance for Reporting Involvement of Patients and the Public
ICS: Integrated Care System
KM: knowledge mobilization
MSK: musculoskeletal
NHS: National Health Service
NIHR: National Institute for Health and Care Research
NT: non–Twitter user
SAG: Stakeholder Advisory Group
T: Twitter user
