# Supplementary material for: Using Twitter (X) to Mobilize Knowledge for First Contact Physiotherapists: Qualitative Study
Source: J Med Internet Res. 2024 Jul 8;26:e55680. doi: 10.2196/55680 (PMC11263900; doi:10.2196/55680)
Supplement: Multimedia Appendix 1 [file jmir_v26i1e55680_app1.docx]

### Interview topic guides

| **Topic Guide 1 – Twitter Users** | | **Topic Guide 2 – Non-Twitter Users** | |
| --- | --- | --- | --- |
| 1 | To start with, can you tell me a bit about your role? | 1 | To start with, can you tell me a bit about your role? |
| 2 | How are you delivering your care at the moment? | 2 | How are you delivering your care at the moment? |
| 3 | What kinds of information do you tend to use to inform your clinical knowledge and professional development? | 3 | Can you tell me the reasons why you don’t use Twitter? |
| 4 | What do you use Twitter for? | 4 | Can you tell me a bit about what you think about the people and information on Twitter being credible sources? |
| 5 | Why Twitter, over other platforms? | 5 | Away from social media, what kinds of information do you tend to use to inform your clinical knowledge and professional development? |
| 6 | What is it that you can discover on Twitter than you can’t by any other means? | 6 | What do you think about colleagues using information on Twitter to develop their clinical knowledge and professional development? |
| 7 | What kinds of MSK Knowledge can be accessed on Twitter? | 7 | What do you think about Twitter as a place to develop networks and relationships? |
| 8 | (If working in different teams / split roles) how does that affect your use of Twitter? |  |  |
| 9 | What types of people do you follow on Twitter? |  |  |
| 10 | Do you have a particular network of FCPs that you follow? |  |  |
| 11 | How do you identify credible people? |  |  |
| 12 | Do you feel that you join in with conversations, or post information on Twitter? Or do you tend to take a step back and observe? |  |  |
| 13 | There is a lot of information on Twitter. How do you prioritise the information you are looking for? |  |  |
| 14 | Have you ever seen clinical conundrums or case studies discussed on Twitter? |  |  |
| 15 | Does anything worry you about using Twitter? |  |  |
| 16 | I have spoken to patients as part of this project, one point that came up was the opportunity for them to follow clinicians on Twitter. How would you feel about this? |  |  |
| 17 | Has sharing knowledge on Twitter changed for you over the last few years? |  |  |
| 18 | Do you think Twitter offers diverse information? |  |  |
| 19 | What are the key ingredients for a Tweet that will make you take notice and either share, like or comment? |  |  |
| 20 | Is there any information you would like to see more of on Twitter? |  |  |
