# Supplementary material for: Using Twitter (X) to Mobilize Knowledge for First Contact Physiotherapists: Qualitative Study
Source: J Med Internet Res. 2024 Jul 8;26:e55680. doi: 10.2196/55680 (PMC11263900; doi:10.2196/55680)
Supplement: Multimedia Appendix 3 [file jmir_v26i1e55680_app3.docx]

### Positionality statement

My academic background includes a BA (Hons) degree in French, German and European studies followed by professional roles as a Translator, Newspaper Journalist, Magazine Editor, PPIE professional and Knowledge Broker. Inspired by my own personal patient journey I am a volunteer for a national spinal charity. My academic interest in KM research and practice developed from my experience of working creatively with stakeholders to mobilise knowledge and have impact on patient care.

Through discussion with peers, reading and a personal research journal I was able to better understand my own position as a researcher within this study, reflecting on how I conducted myself and interacted with both participants and the data. This helped me to become more aware of how my own professional and personal experiences can influence my research. I have summarised my key reflections below:

- As a journalist I strived to be unbiased and balanced but the intended readership of an article and editorial steer sometimes resulted in writing taking on a certain ‘slant’. I believe that like journalists, exploratory researchers cannot be completely value free and separate their personal perspectives, unconscious biases and experiences. However, I strived to acknowledge these and ensure my research outputs were as unbiased and transparent as possible by mitigating where I could. My experience enabled me to encourage a relaxed rapport with participants for rich data but I did not disclose my former employment.
- As a confident social media user, I became aware and sometimes frustrated that this may not be the case for others.
- I will never fully understand the demands and pressures of working in professional clinical MSK settings. Awareness of this made me proactively try to further my understanding of the FCP role through discussions with FCP and clinical colleagues.
- My positionality influenced what interested me in the data, as a communicator, social media user and patient advocate. I took care to constantly return to the research question and the study team helped me to challenge this and become more neutral and nuanced.
