# Supplementary material for: Using Twitter (X) to Mobilize Knowledge for First Contact Physiotherapists: Qualitative Study
Source: J Med Internet Res. 2024 Jul 8;26:e55680. doi: 10.2196/55680 (PMC11263900; doi:10.2196/55680)
Supplement: Multimedia Appendix 4 [file jmir_v26i1e55680_app4.docx]

### Analysis steps

| **Step** | **Description** |
| --- | --- |
| ***Familiarisation with the dataset*** | A random selection of interviews (conducted between November 2021 and February 2022) was transcribed by LC (qualitative researcher, knowledge mobilisation practitioner), all transcripts were read and re-read and audio recordings listened to. Initial observations and insights were noted in the form of memos. |
| ***Coding*** | Descriptive codes were captured systematically using the comments box function in Word by LC, with double coding of a subset of transcripts by LS (qualitative researcher, knowledge mobilisation research fellow, physiotherapist) and JQ (qualitative researcher, physiotherapist) As analysis progressed codes became more interpretive. Quotes from participants were collected. 1863 codes were copied from Word into an Excel spreadsheet, with each column listing all the codes relevant to each participant. |
| ***Generating initial themes*** | Duplicate and similar codes were deleted or combined, leaving 578 codes. Initial patterns of meaning were then identified from these 578 codes and codes were clustered together into categories that share a particular meaning or idea (central organising concept). This resulted in 19 initial themes being generated. |
| ***Developing and reviewing themes*** | The 19 initial themes were further interpreted and refined into six initial themes through ongoing reflection and discussions with the research team. Thematic maps were developed to clarify and distinguish themes, subthemes and the relationships between them. |
| ***Refining, defining and naming themes*** | The six initial themes, definitions, relationships and boundaries were discussed at a dedicated SAG meeting (held 26.04.22) and a meeting with experienced qualitative research colleagues (held 08.07.22), to support the move away from descriptive to interpretive themes. Participants did not feedback on findings directly. |
| ***Writing up*** | Further analysis took place during the write up phase, whereby the names of the themes were further refined and subthemes were re-organised. A final thematic map was created to illustrate the relationship between these as well as a written summary. |
