# Supplementary material for: Using Twitter (X) to Mobilize Knowledge for First Contact Physiotherapists: Qualitative Study
Source: J Med Internet Res. 2024 Jul 8;26:e55680. doi: 10.2196/55680 (PMC11263900; doi:10.2196/55680)
Supplement: Multimedia Appendix 5 [file jmir_v26i1e55680_app5.docx]

### Patient and Public Involvement

| **Section and topic** | **Item** | **Section where reported and details** |
| --- | --- | --- |
| Aim | Report the aim of PPIE in this study | Aim of PPIE involvement is included in the methods. |
| Methods | Provide a clear description of the methods used for PPIE in this study | Involvement followed the UK Standards for Public Involvement in Research [25] which included financial reimbursement for their time, and support before and after meetings. |
| Study results | Outcomes – report the results of PPIE in the study, including both positives and negative outcomes | Partnership working and co-production ensured mutual understanding between stakeholder groups, and meaningful and collaborative PPIE which provided additional contextual insights into MSK patient care not previously considered (see discussion and conclusion section below). |
| Discussion and conclusion | Outcomes – Comment on the extent to which PPIE influenced this study overall. Describe positive and negative effects | Differing opinions arose in the SAG between FCP and public members, during discussion around patients following and engaging with FCPs on Twitter. Discussion resulted in the inclusion of Q16 in the topic guide: “I have spoken to patients as part of this project, one point that came up was the opportunity for them to follow clinicians on Twitter. How would you feel about this?” This enabled meaningful new knowledge to be co-constructed in interviews as participants had not considered this before. |
| Reflections / critical perspective | Comment critically on the study, reflecting on the things that went well and those that did not, so others can learn from this experience | This study presented a challenge of involving public contributors in a healthcare professional focussed study where contexts and experiences differ. The SAG enabled trusted relationships between both groups to grow and open, plain discussion to be had in a safe space. |
