# Supplementary material for: Using Twitter (X) to Mobilize Knowledge for First Contact Physiotherapists: Qualitative Study
Source: J Med Internet Res. 2024 Jul 8;26:e55680. doi: 10.2196/55680 (PMC11263900; doi:10.2196/55680)
Supplement: Multimedia Appendix 6 [file jmir_v26i1e55680_app6.pdf]

Keele University FMHS Faculty Research Ethics Committee
g.p.j.moss@keele.ac.uk

28th October 2021

Dear Laura

**Project Title:**	Twitter for knowledge mobilisation: Investigating the attitudes, beliefs and behaviours of musculoskeletal (MSK) First Contact Physiotherapists (FCPs)
**REC Project Reference:**	MH-210199
**Type of Application**	Main application

Keele University's Faculty of Medicine and Health Sciences Research Ethics Committee (FMHS FREC) reviewed the above project application.

Final Opinion
Thank you for summarising the amendments in a detailed but extremely clear manner. The FMHS FREC can now recommend that this study receives a **Favourable Ethical Opinion**.

<u>Conditions / recommendations:</u>
There are no **conditions** attached to this application. There are, however, standard reporting requirements to consider, below:

Reporting requirements
The University's standard operating procedures give detailed guidance on reporting requirements for studies with a favourable opinion including:

- Notifying the relevant FREC of substantial amendments to an approved study
- Notifying the relevant FREC of issues which may have an impact upon ethical opinion of the study
- Progress reports, as appropriate
- Notifying the relevant FREC of the end of the study

Documents reviewed
The documents reviewed were:

Document	Version	Date
All documents submitted with MH-210199 including revisions		

Yours sincerely,

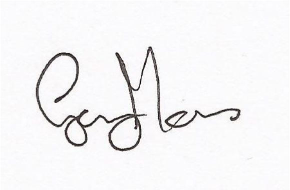

Dr Gary Moss

Chair
